# Development and Internal Validation of the AP-Score for Predicting Percutaneous Nephrostomy Use in Acute Obstructive Pyelonephritis

**DOI:** 10.3390/jcm15176662

**Published:** 2026-08-28

**Authors:** Hakan Şığva, Mehmet Sevim, Arif Mehmet Duran, Muhammed Tosun, Ömer Büyüktepe, Fatih Gökalp

**Affiliations:** 1 Urology Clinic, Van Training and Research Hospital, Van 65300, Turkey; dokterms@gmail.com (M.S.); arifmehmetduran@hotmail.com (A.M.D.); drmuhammedtosun@gmail.com (M.T.); omer_buyuktepe@hotmail.com (Ö.B.); 2 Urology Clinic, Osmaniye Training and Research Hospital, Osmaniye 80000, Turkey; fatihgokalp85@gmail.com

**Keywords:** acute obstructive pyelonephritis, ureteral stone, hydronephrosis, C-reactive protein, serum creatinine, percutaneous nephrostomy, clinical prediction score

## Abstract

**Background/Objectives**: Acute obstructive pyelonephritis (AOP) is a urological emergency requiring urgent decompression. We aimed to develop and internally validate an interpretable admission-based score (AP-Score) to predict the likelihood of undergoing percutaneous nephrostomy (PCN) and to test whether it outperforms hydronephrosis grade alone. **Methods**: We retrospectively analyzed 231 adults with AOP treated with antibiotics alone, double-J stenting, or PCN at a single center. Four admission variables (temperature, C-reactive protein [CRP], serum creatinine, and hydronephrosis grade) were evaluated by multivariable logistic regression and likelihood ratio testing. Discrimination was compared against hydronephrosis grade alone using the DeLong test; calibration and clinical usefulness were assessed, and optimism was estimated using 1000 bootstrap resamples. **Results**: In the likelihood ratio test, while adding CRP to hydronephrosis grade significantly improved model fit (*p* < 0.001), serum creatinine provided a smaller additional contribution (*p* = 0.013). The AP-Score (0–8 points) combined these three variables included in the model; in the categorical model, the grade of hydronephrosis and CRP appeared as independent predictors, whereas creatinine was included due to its contribution on a continuous scale. The score achieved an AUC of 0.830 (95% CI 0.771–0.886), performing significantly better than the model using only the grade of hydronephrosis (AUC 0.772; ΔAUC = +0.058, DeLong *p* = 0.010; IDI = 0.061, *p* = 0.002). The optimism-corrected AUC was calculated as 0.827, and calibration was found to be adequate (Hosmer–Lemeshow *p* = 0.342). A threshold value of ≥4 points yielded a sensitivity of 85.3% and a specificity of 65.0% (OR = 10.79, 95% CI 5.12–22.70); no patients scoring 0–1 points (n = 37) underwent nephrostomy. **Conclusions**: The AP-Score—which combines a structural parameter (degree of hydronephrosis) with two systemic markers (CRP and creatinine)—is a simple and well-calibrated score that performs significantly better than the degree of hydronephrosis alone. The AP-Score was also associated with additional clinical outcomes, suggesting that it captures aspects of the underlying clinical course in addition to local treatment patterns. However, because the primary endpoint reflects the drainage modality selected under a single-center institutional protocol, the score should be considered hypothesis-generating until externally validated in independent, preferably multicenter cohorts with different drainage practices.

## 1. Introduction

Acute obstructive pyelonephritis presents as a serious urological emergency requiring immediate intervention due to its potential to rapidly progress to life-threatening complications, including septic shock [1]. The coexistence of urinary tract obstruction and infection creates a high-pressure environment that facilitates the entry of bacteria into the bloodstream; therefore, early recognition of the condition and prompt decision-making regarding treatment are of vital importance. In clinical practice, physicians must rapidly determine whether the patient can be managed with conservative treatment alone or requires urgent invasive drainage, as delays are associated with poorer clinical outcomes and prolonged hospital stays [2]. Obstructive pyelonephritis can rapidly progress to pyonephrosis—a condition characterized by the accumulation of purulent material within the collecting system—which necessitates urgent intervention to prevent septic shock [3].

The optimal drainage method (e.g., ureteral stenting or percutaneous nephrostomy) often depends on various clinical and imaging parameters, highlighting the need for a reliable predictive tool to guide timely and appropriate treatment [4]. The existing literature emphasizes the critical need for effective predictive models for obstructive pyelonephritis, particularly for identifying patients at risk of severe outcomes (e.g., sepsis) or those who will ultimately undergo a more invasive drainage procedure, such as percutaneous nephrostomy [4]. For instance, data-driven algorithms have been investigated to predict septic shock in obstructive pyelonephritis and to develop diagnostic models for stone-related pyonephrosis; these studies demonstrate growing interest in data-driven approaches aimed at optimizing clinical decision-making processes [5,6].

Most current models focus primarily on predicting mortality or intensive care unit (ICU) outcomes, but not on guiding the initial choice of treatment method [7]. Despite increasing interest in data-driven approaches, there remains a lack of easily interpretable, admission-based clinical scoring systems designed to estimate the likelihood of different treatment approaches, including percutaneous nephrostomy, in patients with acute obstructive pyelonephritis. To address this gap, we developed the AP-Score. The AP-Score integrates admission parameters such as temperature, C-reactive protein (CRP), serum creatinine (Scr), and hydronephrosis grade, which have previously been identified as critical indicators of disease severity and predictors of intervention in similar contexts [8,9]. Similarly, nomograms have been developed to predict sepsis in patients with obstructive pyelonephritis due to urolithiasis, highlighting the utility of predictive tools in guiding interventions [10].

Current evidence indicates that clinical baseline indicators—including neutrophil counts (Neu), serum creatinine (Scr), and imaging findings such as the severity of hydronephrosis (HD)—are critical and independent risk factors for identifying these high-risk cases [11].

In this context, combining the variables in question into a single composite score could assist clinicians in making faster and more consistent decisions at the bedside [12]. However, a critical point is that the use of a composite score is justified only if it performs better than its strongest individual component. Therefore, we designed our study not only to create such a score but also to formally compare and test it against the grade of hydronephrosis alone.

## 2. Materials and Methods

### 2.1. Study Design and Population

This retrospective observational cohort study was conducted at a tertiary referral center to evaluate treatment classification in patients diagnosed with acute obstructive pyelonephritis (AOP). The AP-Score—a simplified clinical scoring system based on admission data—was developed to predict PCN use within the institutional drainage pathway.

Consecutive adult patients admitted between January 2022 and November 2025 were included in the study. Ethical approval was obtained from the Clinical Research Ethics Committee of the University of Health Sciences, Van Training and Research Hospital (Approval No.: GOKAEK/2026-04-08 date: 10 April 2026). Due to the retrospective design of the study, obtaining informed consent was not required.

Patients over the age of 18 with a clinical diagnosis of acute obstructive pyelonephritis (AOP) and imaging-confirmed obstruction were included in the study. Pregnant or pediatric patients, those with pyelonephritis not associated with obstruction, and patients with incomplete baseline laboratory or radiological data were excluded. The study cohort consisted of 231 patients. Based on the final treatment method administered, patients were categorized into three groups: antibiotic therapy alone (AB, n = 37), double-J stent placement (DJ, n = 126), and percutaneous nephrostomy (PCN, n = 68). Patients who underwent both percutaneous nephrostomy and subsequent double-J stent placement were analyzed within the PCN group, as nephrostomy was the initial invasive drainage method employed. A standard institutional decompression protocol was followed; this protocol designated retrograde double-J stent placement as the first-line drainage method, while reserving percutaneous nephrostomy for cases where retrograde access failed or was technically unfeasible, or where the patient was hemodynamically unsuitable for the retrograde approach. Retrograde drainage capabilities were available throughout the study period.

The diagnosis of AOP was established based on the presence of the following [1]:

- Systemic inflammatory response (fever > 38 °C or elevated inflammatory markers);
- Localized symptoms (flank pain or costovertebral angle tenderness);
- Pyuria confirmed by urinalysis;
- Radiological findings of upper urinary tract obstruction.

### 2.2. Data Collection and Imaging

To ensure real-world clinical applicability, only variables available at the time of hospital admission were recorded. These included demographic characteristics, comorbidities, vital signs, laboratory parameters, and radiological findings.

The severity of hydronephrosis was classified according to the Society for Fetal Urology (SFU) grading system using non-contrast CT images obtained at admission. Although the SFU system was originally developed for grading pediatric hydronephrosis on ultrasonography [13], hydronephrosis severity has also been assessed using non-contrast CT in adult populations [14]. We adopted the SFU framework because of its simplicity and ease of application to routinely reported imaging findings, while acknowledging that it has not been formally validated for adult CT. Hydronephrosis grade was obtained from the reporting radiologist’s routine admission CT report, which was completed before the definitive drainage decision. No study-specific blinding procedure was implemented. Because the study relied on these original radiology reports, formal inter-reader agreement could not be assessed, and this was considered a limitation.

Stone characteristics were obtained from non-contrast CT reports generated at the time of hospital admission. Stone location was classified as upper urinary tract (intrarenal calyces, renal pelvis, ureteropelvic junction, or proximal ureter), mid-ureter, or distal ureter/ureterovesical junction; in cases with multiple stones, the most proximal level causing obstruction was used as the reference point. The added value of stone location (entire cohort) and stone size (available subgroup) compared to the AP-Score was evaluated using the likelihood ratio test. Procalcitonin levels were recorded in only 20 patients (8.7%) and thus could not be included in the analysis as a candidate variable; other novel inflammatory indices (e.g., delta neutrophil index) were not available in this retrospective dataset.

To address the risk associated with data-driven threshold selection, two pre-specified sensitivity analyses were conducted. First, the CRP categories used in the score—including tertiles derived from cohort data—were modified using seven different sets of threshold values, and the AUC was recalculated for each scenario. Second, pairwise *post hoc* comparisons between treatment groups were performed using the Mann–Whitney U test and the Bonferroni correction for multiple testing.

### 2.3. Treatment Strategies

Patients were classified as follows:

- Antibiotic therapy alone;
- Double-J ureteral stenting;
- Percutaneous nephrostomy.

Empiric intravenous antibiotic therapy was initiated immediately after culture samples were obtained. While third-generation cephalosporins were used as first-line therapy, carbapenems were reserved for patients presenting with sepsis or hemodynamic instability. Once the results were available, antibiotic therapy was adjusted based on the findings of urine cultures and antibiograms.

DJ stent placement was performed cystoscopically under regional or general anesthesia. Percutaneous nephrostomy (PCN) was primarily performed in the prone position, under ultrasound and fluoroscopy guidance, using 8–10 Fr catheters. Both procedures were provided as part of a 24 h emergency service. The median time from hospital admission to the clinical decision to perform urinary decompression (i.e., the time at which the treating team documented the indication for drainage) was 6 h (range: 3–12). This is distinct from the time to the drainage procedure itself, reported in the Results. The timing of the intervention (daytime or nighttime) did not significantly influence the choice of drainage method.

The AP-Score was derived empirically from the study data. Initially, all four candidate variables (body temperature, CRP, serum creatinine level, and hydronephrosis grade) were included in a multivariable logistic regression model designed to predict PCN use within the institutional drainage pathway. Variables that did not make an independent contribution were excluded from the model, and this exclusion was validated using the likelihood ratio test. Subsequently, integer-based point weights were assigned to the remaining variables in proportion to the direction and relative magnitude of their regression coefficients: the highest weight was assigned to the grade of hydronephrosis—the most dominant predictor (Grade 1/2/3 = 0/2/4 points)—while the two laboratory markers were scored across three ordinal categories (0/1/2 points). We deliberately chose to use simple, rounded weights rather than a coefficient-based scoring algorithm (such as the Sullivan method) because, in this cohort, a purely coefficient-based system resulted in an impractically wide score range without enhancing discriminative power. In contrast, this simpler method maintained discriminative power while ensuring the score could be easily applied at the bedside.

Total AP Score = Hydronephrosis score + CRP score + Creatinine score

Total score range: 0–8

Risk categories:

- Low risk (0–1);
- Intermediate risk (2–3);
- High risk (≥4).

No patients in the low-risk group (0–1 points) underwent percutaneous nephrostomy. In the intermediate-risk group (2–3 points), the most frequently performed procedure was double-J stent placement. In the high-risk group (≥4 points), however, the most common method employed was percutaneous nephrostomy.
**Variable****Category****Points**Hydronephrosis gradeGrade 1/Grade 2/Grade 30/2/4CRP at admission (mg/L)<50/50–150/>1500/1/2Serum creatinine (mg/dL)<1.2/1.2–2.0/>2.00/1/2

Temperature was evaluated but did not provide independent information (likelihood ratio *p* = 0.772) and is not part of the final score.

### 2.4. Statistical Analysis

The normality of continuous variables was assessed using the Shapiro–Wilk test. Variables exhibiting a normal distribution (age) were presented as mean ± standard deviation and compared using one-way ANOVA, whereas variables not exhibiting a normal distribution were presented as median (min–max) and compared using the Kruskal–Wallis test. Categorical variables were analyzed using the chi-square test or Fisher’s exact test, as appropriate. Pairwise post hoc comparisons were performed using the Mann–Whitney U test with Bonferroni correction.

Model development and reporting were conducted in accordance with the TRIPOD (Transparent Reporting of a multivariable prediction model for Individual Prognosis Or Diagnosis) statement for prediction model studies [15]. As the AP-Score is a traditional logistic regression-based score that does not employ machine learning or artificial intelligence methods, the TRIPOD+AI (2024) extension was not applicable to this study; nevertheless, the study adhered to shared principles such as transparent handling of variables, internal validation, and calibration reporting.

The performance of the AP-Score in predicting PCN use was evaluated using receiver operating characteristic (ROC) curve analysis. Based on the principle that a composite score is clinically justified only if it outperforms its strongest individual component, the ROC curve of the AP-Score was formally compared—using the DeLong test for correlated ROC curves—with that of hydronephrosis grade alone and with an alternative model comprising hydronephrosis grade and CRP without creatinine.

Multivariable binary logistic regression analysis was performed to identify independent predictors of PCN use among four candidate variables. Nested models were compared using the likelihood ratio test to determine which variables could be excluded from the model without loss of information. Odds ratios (OR) and 95% confidence intervals (CI) were calculated, and the optimal threshold value was determined using the Youden index. Model calibration was assessed graphically via calibration plots and quantitatively using the calibration slope, overall calibration intercept, and the Hosmer–Lemeshow goodness-of-fit test. Since the calibration slope in the dataset from which the model was derived is structurally 1.0, we report the optimism-corrected calibration slope—obtained via the bootstrap method—which also serves as a uniform shrinkage factor. During the internal validation process, 1000 bootstrap resamples were used, repeating all derivation steps—including coefficient estimation and integer score assignment—to obtain optimism-corrected estimates for the area under the curve (AUC) and the calibration slope. The added value of the AP-Score compared to the grade of hydronephrosis alone was evaluated using the DeLong test for the associated ROC curves, the integrated discrimination improvement (IDI), and the category-free net reclassification index (NRI). Clinical utility was assessed via decision curve analysis (DCA), comparing the net benefit of the AP-Score across a range of threshold probabilities against the net benefit of the grade of hydronephrosis alone and the default strategies of performing nephrostomy on either all or no patients. A *p*-value of less than 0.05 was considered statistically significant.

## 3. Results

### 3.1. Study Population and Clinical Outcomes

In all patients (100%), the underlying cause of the obstruction was a ureteral stone; no cases of extrinsic malignant compression were encountered in the study group.

The median length of hospital stay was longest in the PCN group (PCN: 9.0 (2.0–30.0) days; DJ: 5.0 (1.0–16.0) days; AB: 5.0 (2.0–10.0) days; *p* < 0.001). The median time from hospital admission to the procedure was similar between the PCN and DJ groups (24.0 (4.0–96.0) and 24.0 (2.0–120.0) hours, respectively); however, the overall distribution differed significantly, with fewer prolonged delays observed in the PCN group (*p* = 0.005). The in-hospital mortality rate was 1/231 (0.4%), and 7 patients (3.0%) required intensive care admission. The 30-day readmission rates were similar across the groups (PCN: 13.4%; DJ: 6.3%; AB: 5.4%; *p* = 0.185).

### 3.2. Baseline Characteristics and Laboratory Findings

Baseline characteristics are summarized in Table 1. Patients who underwent percutaneous nephrostomy presented with significantly higher grades of hydronephrosis, higher baseline CRP, and higher creatinine levels (HN: *p* < 0.001; CRP: *p* < 0.001; creatinine: *p* = 0.001). Body temperature also differed between the groups (*p* = 0.010). Stage 3 hydronephrosis was most frequently observed in the PCN group (52.9%; 36/68) compared to the DJ group (9.5%; 12/126) and the AB group (0.0%; 0/37).

The final AP-Score assigns points for hydronephrosis grade, CRP and creatinine, yielding a total range of 0–8 (Table 2A). The scoring system revealed a stepwise shift in treatment strategy across the three risk groups (Table 2B). In the low-risk group (0–1 points, n = 37), double-J stent placement or antibiotic therapy was preferred, and no patients underwent percutaneous nephrostomy. In the intermediate-risk group (2–3 points, n = 79), double-J stent placement (68.4%) was the predominant method, with a PCN use rate of 12.7%. In the high-risk group (≥4 points, n = 115), however, PCN was the most frequently performed procedure (50.4%) (*p* < 0.001). The predictive performance of the AP-Score at the ≥4-point threshold for PCN use is presented in Table 2. The rate of percutaneous nephrostomy was significantly higher in patients with a score of ≥4 points compared to those below the threshold (OR = 10.79; 95% CI: 5.12–22.70; *p* < 0.001). The fact that none of the 37 patients with scores of 0–1 underwent nephrostomy (0%; 95% CI 0–9.4%, Wilson) supports the score’s utility as a tool for exclusion at the lower end of the range.

### 3.3. Model Development and Multivariable Analysis

When all four candidate variables were simultaneously included in the model, the grade of hydronephrosis and the CRP level at presentation were identified as the strongest predictors of PCN use. A sequential likelihood ratio test—using each variable in its continuous (numerical) form to avoid artifacts arising from categorization—demonstrated that adding CRP to the hydronephrosis grade significantly improved model fit (χ^2^ = 19.43; df = 1; *p* < 0.001); that adding serum creatinine level yielded a further significant, albeit smaller, improvement (χ^2^ = 6.21; df = 1; *p* = 0.013); and that the subsequent addition of body temperature did not improve model performance (χ^2^ = 0.08; df = 1; *p* = 0.772). Consequently, body temperature was excluded from the model. In the final model utilizing categorical score components (Table 3), the grade of hydronephrosis (OR = 7.50; 95% CI 4.03–13.96; *p* < 0.001) and CRP (OR = 2.48; 95% CI 1.58–3.88; *p* < 0.001) were identified as independent predictors.

Serum creatinine warrants explicit comment, as its role differs by scale. On likelihood ratio testing in continuous form, creatinine contributed independent prognostic information beyond hydronephrosis grade and CRP (*p* = 0.013), which is why it was retained in the score. When collapsed into three ordered categories for bedside scoring, its adjusted association was attenuated and no longer statistically significant (OR = 1.40, 95% CI 0.84–2.34, *p* = 0.199). This attenuation may partly reflect the loss of information and statistical power associated with categorizing a continuous variable, rather than necessarily indicating that creatinine is uninformative. We retained creatinine for three reasons: its independent contribution on the continuous scale, its universal and immediate availability at admission, and its physiological plausibility as a marker of obstruction-related renal impairment. We report the categorical odds ratio transparently so that readers can weigh this trade-off; a two-variable version of the score remains a reasonable alternative for settings that prefer maximal parsimony. Future studies may also explore modeling creatinine as a continuous variable or using spline transformations to preserve its full predictive information within a scoring framework.

### 3.4. Discrimination, Model Comparison and Calibration

**Figure 1:**
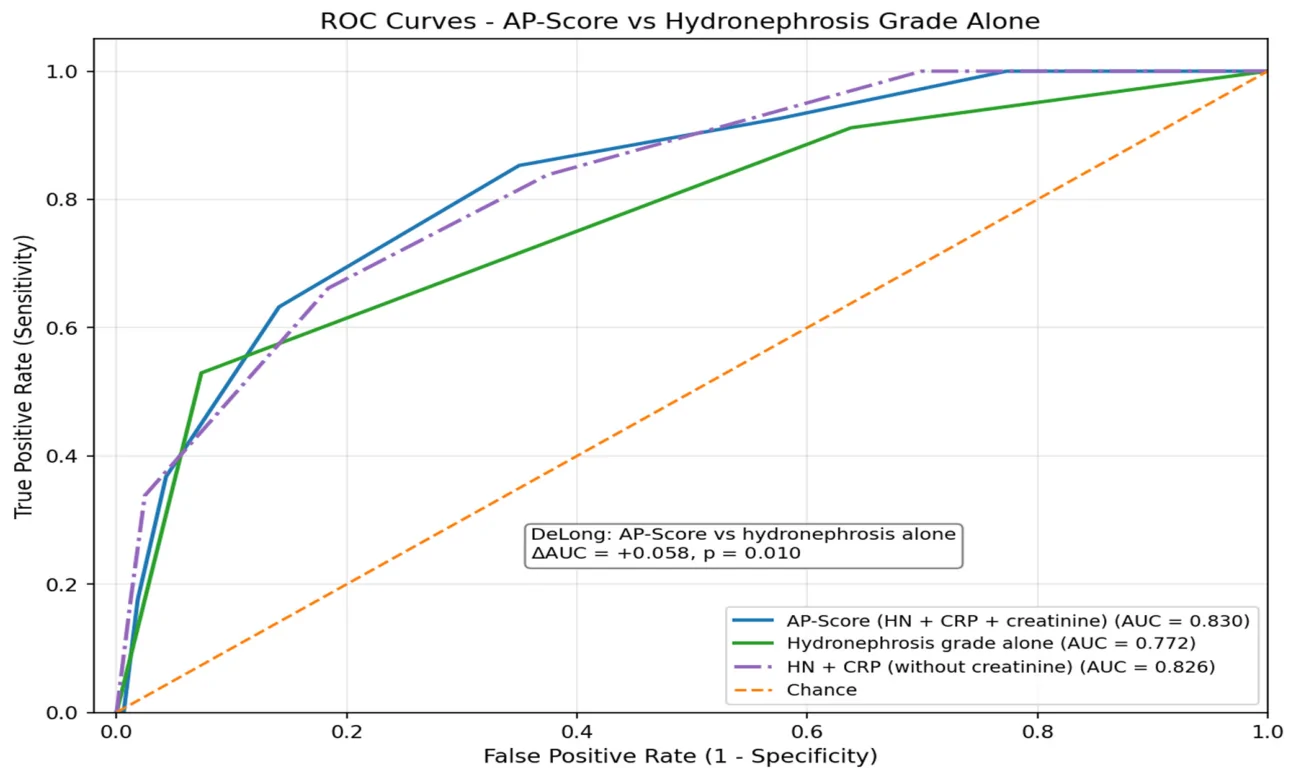
ROC curves for the AP-Score, hydronephrosis grade alone, and the hydronephrosis + CRP model without creatinine for predicting PCN use. AP-Score, Acute Pyelonephritis Score; ROC, receiver operating characteristic; AUC, area under the curve; PCN, percutaneous nephrostomy; CRP, C-reactive protein.

The AP-Score demonstrated good discriminative performance for predicting PCN use, with an area under the curve (AUC) of 0.830 (95% CI: 0.771–0.886) (Figure 1). Internal validation using 1000 bootstrap resamples—in which the entire derivation process was repeated for each sample—yielded an optimism-corrected AUC of 0.827, indicating minimal overfitting.

Notably, the AP-Score alone performed significantly better than the hydronephrosis grade (AUC 0.830 vs. 0.772; ΔAUC = +0.058; DeLong *p* = 0.010), and this added value was confirmed by an integrated discrimination improvement of 0.061 (95% CI 0.023–0.098, *p* = 0.002) (Table 4). A threshold value of ≥4 points provided the optimal balance between sensitivity and specificity (Youden index = 0.503), yielding a sensitivity of 85.3% (95% CI 75.0–91.8%), specificity of 65.0% (95% CI 57.4–71.9%), positive predictive value of 50.4% (95% CI 41.4–59.4%), and negative predictive value of 91.4% (95% CI 84.9–95.3%).

The observed PCN rate showed a monotonic increase across the score values, rising from 0% in the 0–1 point range to 76% and 86% at scores of 6 and 7, respectively. Higher AP-Scores were also associated with a significantly prolonged hospital stay (*p* < 0.001), providing supportive evidence that the score is also associated with the underlying clinical course beyond its association with the drainage modality selected.

### 3.5. Association with Additional Clinical Outcomes

Since the primary endpoint—the drainage modality selected by the treatment team—may be influenced by institutional practices as well as disease severity, we additionally examined the associations of the AP-Score with other clinical outcomes. The score correlated with the length of hospital stay (Spearman rho = 0.451, *p* < 0.001), the time to defervescence (rho = 0.353, *p* < 0.001), and the time to clinical stabilization (rho = 0.315, *p* < 0.001). The median length of stay showed a monotonic increase, ranging from 3 days at a score of 0 to 9 days at a score of 6.

Notably, these associations persisted even after adjustments were made for the treatment regimen actually administered. In multivariable linear models that included the treatment group, each additional point on the AP-Score was independently associated with a 0.70-day increase (*p* < 0.001) in the duration of hospitalization and a 4.76 h increase (*p* < 0.001) in the time to defervescence; however, the treatment group itself was not a significant predictor of the time to defervescence (DJ: *p* = 0.195; PCN: *p* = 0.579) (Table 5). A composite serious outcome endpoint (sepsis, ICU admission, or in-hospital death) occurred in only 8 patients (3.5%). Although the event rate rose from 0% in patients with a score of 0–1 to 5.3% in those with a score of ≥4, this analysis lacked sufficient statistical power, and the observed association did not reach statistical significance (OR per point 1.46; 95% CI 0.93–2.28; *p* = 0.099). These findings provide supportive evidence that the AP-Score captures aspects of the underlying clinical course in addition to its association with the drainage modality selected; however, they do not establish independence from clinician-selected treatment.

### 3.6. Calibration, Reclassification and Clinical Usefulness

**Figure 2:**
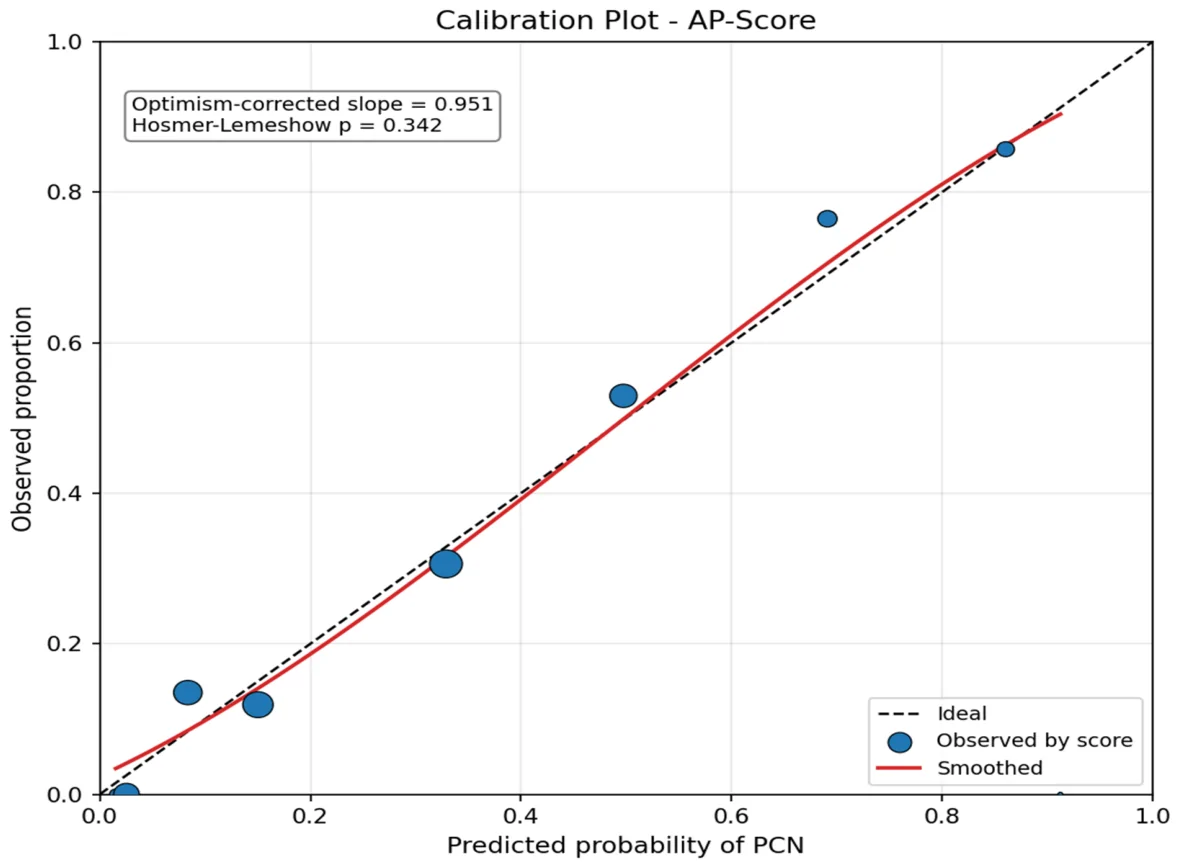
Calibration Plot of the AP-Score for Predicting Percutaneous Nephrostomy Use AP-Score, Acute Pyelonephritis Score; PCN, percutaneous nephrostomy.

Calibration was adequate across the entire predicted risk range (Figure 2). The Hosmer–Lemeshow test was not statistically significant (χ^2^ = 3.34, df = 3, *p* = 0.342). As the observed calibration slope in the development sample was 1.0, we report the optimism-corrected slope value obtained via the bootstrap method; this value is 0.951—close to unity (1.0)—indicating minimal shrinkage. Observed PCN rates increased monotonically with rising scores, ranging from 0% in the 0–1 score range to over 75% in the 6–7 score range.

Reclassification analysis confirmed the added value of incorporating CRP and creatinine levels alongside the grade of hydronephrosis. The Integrated Discrimination Improvement (IDI) value was determined to be 0.061 (95% CI 0.023–0.098; *p* = 0.002); this value reflected a higher mean predicted risk level in patients who underwent nephrostomy and a lower level in those who did not. Given the known statistical limitations of category-free NRI, we rely on the DeLong test and IDI as the primary evidence for this added value.

**Figure 3:**
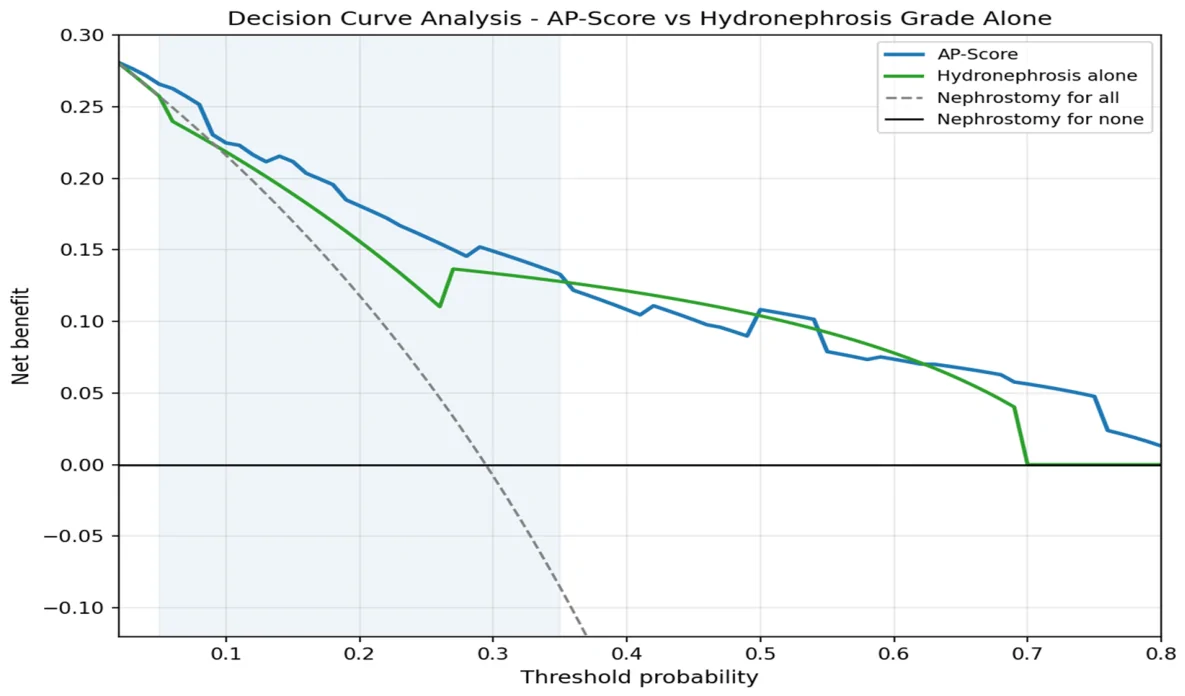
Decision Curve Analysis Comparing the AP-Score with Hydronephrosis Grade Alone DCA, decision curve analysis; AP-Score, Acute Pyelonephritis Score.

Decision curve analysis (Figure 3) demonstrated that, within a threshold probability range of approximately 5–35%, the AP-Score yielded a higher net benefit compared to the grade of hydronephrosis alone, the strategy of performing nephrostomy on all patients, and the strategy of performing nephrostomy on no patients. This range is relevant for clinicians wishing to avoid overlooking a patient with a higher likelihood of undergoing PCN within the institutional pathway, and it corresponds to the score’s utility for “rule-out” purposes. At higher threshold values, however, the curves converged.

### 3.7. Stone Characteristics and Sensitivity Analyses

Since stone characteristics could be reasonable predictors of the drainage method, we investigated whether they provided information beyond the AP-Score. Data on stone location were available for 225 patients (97.4%): upper tract in 121 (53.8%), mid-ureter in 36 (16.0%), and distal ureter/ureterovesical junction (UVJ) in 68 (30.2%). The rate of percutaneous nephrostomy (PCN) did not differ significantly by location (upper 33.1%, mid 33.3%, distal 22.1%; *p* = 0.249), and adding stone location to the AP-Score model did not improve model fit (likelihood ratio χ^2^ = 0.98, df = 2, *p* = 0.612; AUC from 0.818 to 0.825). Data on maximum stone diameter were available for 58 patients (25.1%). In this subgroup, stones were larger in patients who underwent PCN (median 10.0 vs. 8.0 mm, *p* = 0.016); however, this association lost significance after adjusting for the grade of hydronephrosis and CRP levels (OR 1.08 per mm increase, 95% CI 0.94–1.24, *p* = 0.291; likelihood ratio *p* = 0.272). This suggests that the association of stone size observed in the univariable analysis may be partly accounted for by the degree of hydronephrosis. Density in Hounsfield units was not routinely recorded and therefore could not be evaluated (Table 6).

To rule out the possibility that the CRP threshold values were selected to maximize apparent performance, the full AP-Score (including creatinine) was recalculated using seven different sets of alternative CRP thresholds. AUC values remained essentially unchanged across all sets, ranging from 0.816 to 0.830 (selected thresholds <50/50–150/>150: AUC 0.830; tertiles derived from cohort data: 0.825) (Table 6). Thus, discriminative power is not sensitive to the precise CRP threshold values; consequently, rounded and clinically conventional values were retained to facilitate ease of use at the point of care.

Post hoc pairwise comparisons (with Bonferroni correction) revealed that the intergroup differences shown in Table 1 stemmed from the PCN group. While CRP values did not differ significantly between the AB and DJ groups (adjusted *p* = 0.444), they were significantly higher in the PCN group compared to both other groups (*p* = 0.002 vs. AB; *p* = 0.003 vs. DJ). The same pattern was observed for the grade of hydronephrosis (*p* = 0.083 between AB and DJ; *p* < 0.001 between PCN and the others), creatinine levels, and body temperature.

The median time from hospital admission to the actual initial drainage procedure (the time the intervention was performed) was 24 h (Interquartile Range [IQR]: 18–48); 57.9% of the procedures were performed within the first 24 h, while 16.2% were performed after more than 48 h. Drainage procedures were performed earlier in patients with sepsis (median 12 h vs. 24 h, *p* = 0.080) and in those with Grade 3 hydronephrosis (*p* = 0.047), indicating that disease severity was a primary factor in determining the urgency of the intervention. The time to intervention was not found to be associated with the length of hospital stay, either in the unadjusted analysis (Spearman rho = −0.096, *p* = 0.182) or after adjustment for hydronephrosis grade and CRP levels (β = +0.010 days/hour, *p* = 0.275).

AP-Score AUC = 0.830 (95% CI: 0.771–0.886) vs. hydronephrosis grade alone AUC = 0.772 (DeLong *p* = 0.010). Optimal cut-off: ≥4 points (Sensitivity: 85.3%, Specificity: 65.0%).

Point size is proportional to the number of patients at each score value. Optimism-corrected calibration slope = 0.951; Hosmer–Lemeshow *p* = 0.342.

The AP-Score yields the highest net benefit across threshold probabilities of approximately 5–35%, the range corresponding to its rule-out use. At higher thresholds, the curves converge.

This roadmap outlines the likelihood of nephrostomy observed within each stratum. It does not mandate any specific treatment method; the scoring system in question was developed based on treatments preferred by clinicians and requires external validation before being used as a decision-making tool.

## 4. Discussion

The AP-Score, developed to estimate the probability of PCN use within our institutional drainage pathway, demonstrated good discrimination in patients with acute obstructive pyelonephritis. The score was derived from multivariable regression and likelihood-ratio testing and subsequently simplified using rounded integer weights for bedside application. We evaluated the score by specifically comparing it with its strongest single component: the grade of hydronephrosis. Ultimately, hydronephrosis remained the most critical predictor—a finding consistent with the clinical reality that antibiotics alone cannot treat severe obstructions. Nevertheless, the inclusion of baseline CRP levels in the model made a significant and meaningful contribution to the score’s accuracy.

The AP-Score significantly outperformed hydronephrosis grade alone (AUC 0.830 vs. 0.772; ΔAUC = +0.058; DeLong *p* = 0.010). An alternative model comprising hydronephrosis grade and CRP without creatinine achieved a similar AUC of 0.826, indicating that creatinine provided only a modest incremental contribution in the simplified categorical formulation. Nevertheless, creatinine was retained because it contributed significantly when modeled continuously (likelihood ratio *p* = 0.013), is routinely available at admission, and has physiological plausibility as a marker of obstruction-related renal impairment. Hydronephrosis is a direct structural indicator of impaired drainage and elevated intrapelvic pressure; since high-grade obstruction reduces the feasibility of retrograde stent placement, antegrade decompression is often preferred in cases of severe edema or impacted stones [16,17]. CRP incorporates information regarding systemic inflammation into the process—data that an anatomical assessment alone cannot capture.

It has been reported that each hour of delay in the decompression of AOP increases the length of hospital stay by approximately 8% [17]. The fact that the length of stay was longer in the PCN group, despite the shorter time to intervention, is likely attributable to the greater severity of the obstruction at the time of presentation rather than the drainage method itself; the scoring system in question aims to identify such patients at an early stage.

This scoring system combines three admission variables into a 0-to-8-point scale, demonstrating an AUC of 0.830 and adequate calibration (Hosmer-Lemeshow *p* = 0.342). General sepsis scores such as qSOFA [18,19] were not designed to guide drainage management and do not incorporate disease-specific anatomical parameters.

Calibration was good (optimism-corrected slope of 0.951), and observed nephrostomy rates showed a steady increase as score values rose. Decision curve analysis revealed a clear benefit compared to using the grade of hydronephrosis alone, particularly at low threshold probabilities (approximately 5–35%), whereas this difference narrowed at higher thresholds. Thus, the additional benefit provided by CRP is concentrated in the range where the clinician is least willing to risk overlooking a patient with a higher likelihood of undergoing PCN within the institutional pathway; this aligns with the score’s relatively high sensitivity and negative predictive value.

Stone characteristics might be expected to determine the drainage strategy, so we examined them directly. Stone location was available for 97.4% of patients and was not associated with PCN use (upper tract 33.1%, mid ureter 33.3%, distal ureter 22.1%; *p* = 0.249); adding it to the model produced no improvement (likelihood ratio χ^2^ = 0.98, df = 2, *p* = 0.612). Maximal stone diameter, available in 25.1%, was larger in patients who underwent PCN on univariable testing (median 10.0 vs. 8.0 mm, *p* = 0.016) but lost significance after adjustment for hydronephrosis grade and CRP (OR 1.08 per mm, 95% CI 0.94–1.24, *p* = 0.291). The effect of stone burden may therefore be partly reflected by the degree of obstruction captured by hydronephrosis grade; Hounsfield density was not routinely reported and could not be assessed.

Two groups of excluded variables warrant separate consideration. Procalcitonin, the delta neutrophil index, and quantitative CT attenuation (Hounsfield units) have yielded promising results in similar clinical contexts. Data on procalcitonin were available for only 20 patients (8.7%), and no difference was observed in this subgroup according to PCN use (median 0.91 vs. 1.00 µg/L; *p* = 0.817); meanwhile, Hounsfield units and the delta neutrophil index were not recorded. The AP-Score was derived from variables universally available at the time of hospital admission and is intended to serve as a practical point-of-care tool rather than relying on an advanced biomarker panel. Whether procalcitonin or stone density would enhance the score’s performance is a question that requires investigation in future studies.

Although retrograde stenting is a more common first-line procedure at many centers, the primary endpoint in this study was percutaneous nephrostomy (PCN) rather than urgent drainage in general. Accordingly, the present model should be interpreted as estimating the probability of PCN use within our institutional pathway rather than a protocol-independent physiological need for nephrostomy. The applicability of this prediction across centers with different drainage practices and thresholds requires external validation.

The CRP threshold values (<50, 50–150, >150 mg/L) were rounded to facilitate ease of use at the bedside rather than being optimized based on the AUC; the fact that AUC values ranged only from 0.816 to 0.830 across seven different sets of thresholds—including tertiles derived from cohort data—indicates that there was no overfitting of the threshold values. The incremental contribution over the grade of hydronephrosis (ΔAUC 0.058) is modest, and the primary practical implication of this approach is the clear identification of a low-risk group.

Upper urinary tract decompression can be achieved via percutaneous nephrostomy under radiological guidance, particularly when anesthesia is poorly tolerated or retrograde stent placement fails [20]. Nephrostomy is also utilized in cases of pyonephrosis and can provide access for the treatment of staghorn calculi [16,17]. While benign intrinsic obstructions can generally be managed with stenting, antegrade drainage may be preferred in cases of extrinsic compression [21,22,23].

Although systematic reviews report similar success rates for both techniques, urinary discomfort may occur more frequently following stent placement [24,25]. The more frequent preference for nephrostomy in high-risk patients in this study aligns with findings indicating that antegrade drainage provides more reliable decompression in cases of severe obstruction [26,27].

Early warning scores such as NEWS, SOFA, qSOFA, and SIRS demonstrate acceptable performance in detecting clinical deterioration in severe urinary tract infections [7,19,28]; meanwhile, scores specific to emphysematous pyelonephritis—despite focusing on mortality [29,30,31]—underscore the importance of early assessment. Urolithiasis, the underlying cause in all of our patients, has been reported to aggravate the severity of emphysematous pyelonephritis [32]. This study differs from others in that it aims to classify treatment rather than predict clinical outcomes.

In the categorical model, CRP remained statistically significant whereas the association for categorized creatinine was attenuated; however, creatinine provided significant incremental information when modeled continuously, supporting its retention in the score.

### Clinical Implications

**Figure 4:**
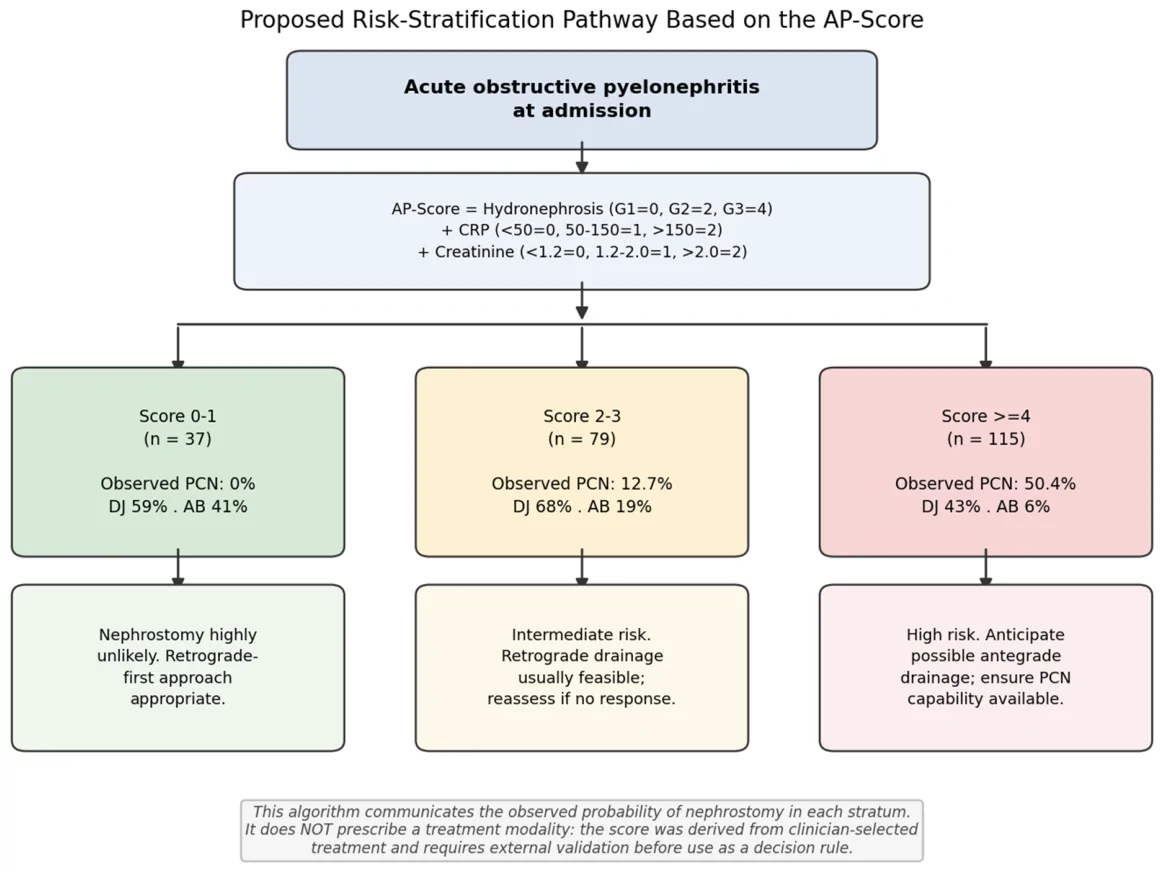
Proposed Risk-Stratification Pathway Based on the AP-Score AP-Score, Acute Pyelonephritis Score; PCN, percutaneous nephrostomy; DJ, double-J stent; AB, antibiotic therapy alone; NPV, negative predictive value; OR, odds ratio.

This scoring system offers the greatest utility at the lower end of the range: No patient scoring 0–1 points underwent PCN (Figure 4), and the negative predictive value at the ≥4-point threshold was 91.4%. Consequently, given the positive predictive value of 50.4%—which implies substantial uncertainty at values above the threshold—the system is more effective at identifying patients with a low likelihood of undergoing PCN within the institutional pathway than at predicting PCN use at higher score values. Developed based on data from a single cohort, this tool complements previous studies addressing predictors of conservative treatment failure in obstructive infections; it should be viewed not as a standalone criterion, but rather as a decision-support tool for hypothesis generation [33].

There are several limitations. First, this was a retrospective, single-center study in which the score was both derived and internally validated. Bootstrap resampling replicated the entire derivation process and estimated the optimism to be only 0.003. Nevertheless, internal validation cannot replace the external validation required before clinical implementation. Second, the primary endpoint—the drainage modality selected by the treating team—reflects both disease severity and clinical decision-making under the institutional treatment pathway. Importantly, the choice between double-J stenting and nephrostomy is also influenced by pre-procedural and patient-specific factors that were not captured in the model—including anesthetic feasibility, ability to tolerate the lithotomy position, anticipated technical feasibility of retrograde access, suitability for prone positioning, and local availability and expertise for each procedure. Because these determinants were not incorporated, the AP-Score should be interpreted as predicting the selection of nephrostomy under our institutional practice pattern rather than an objective, protocol-independent requirement. The association of the AP-Score with additional clinical outcomes, specifically length of hospital stay and time to fever resolution, persisted after adjustment for the intervention performed (Table 5), providing supportive evidence that the score also captures aspects of the underlying clinical course. However, these findings do not eliminate potential circularity because the primary endpoint remains a clinician-selected treatment. In addition, local drainage practices—retrograde stenting as the initial approach, followed by nephrostomy when retrograde access fails or is not feasible—may differ across institutions.

External validation is therefore the essential next step. Ideally, the AP-Score should be tested in an independent, preferably multicenter cohort of patients with acute obstructive pyelonephritis, applying the identical point definitions without re-derivation. Such a study should assess discrimination (AUC), calibration (calibration slope and intercept, with recalibration if required), and clinical usefulness (decision curve analysis) in the new setting and should ideally include centers with different stenting-versus-nephrostomy thresholds to evaluate the generalizability of the protocol-dependent endpoint. Prospective data collection would additionally allow standardized timing definitions and the inclusion of biomarkers not routinely available in the present cohort.

Finally, a direct comparison with established sepsis scores such as qSOFA or MEDS could not be performed because several of their component variables were not consistently available in this retrospective dataset; prospective studies should include these comparators.

## 5. Conclusions

We propose the AP-Score, a three-variable score based on admission hydronephrosis grade, C-reactive protein, and serum creatinine, to support early risk stratification in acute obstructive pyelonephritis. Hydronephrosis grade and CRP were independent predictors of nephrostomy selection under our institutional protocol, while creatinine was retained because of its significant contribution when modeled continuously. The score significantly improved discrimination over hydronephrosis grade alone, and no patient scoring 0–1 underwent PCN in this cohort. Because the endpoint was the drainage modality selected under a single-center institutional protocol, the AP-Score should be interpreted as estimating the probability of undergoing PCN within this pathway rather than an absolute, protocol-independent patient need. Its association with additional clinical outcomes provides supportive evidence that it captures aspects of the underlying clinical course, but does not eliminate the potential circularity inherent in a clinician-selected treatment endpoint. These findings should therefore be regarded as hypothesis-generating and require external validation, preferably in multicenter cohorts with different drainage practices, before clinical adoption.

## Figures and Tables

**Table 1:** Baseline Characteristics According to Treatment Group.

Parameter	AB (n = 37)	DJ (n = 126)	PCN (n = 68)	*p*-Value	Post hoc (Bonferroni)
Age (years)	47.2 ± 15.4	49.6 ± 15.2	53.3 ± 17.3	0.128	—
Sex (Male)	25 (67.6%)	101 (80.2%)	51 (75.0%)	0.263	—
Diabetes Mellitus (%)	5 (13.5%)	18 (14.3%)	14 (20.6%)	0.470	—
Creatinine (mg/dL)	1.23 (0.6–1.9)	1.39 (0.6–7.3)	1.64 (0.2–9.7)	**0.001**	**AB vs. PCN 0.002; DJ vs. PCN 0.049; AB vs. DJ ns**
CRP (mg/L)	29.0 (2.0–298.0)	59.0 (2.0–371.0)	112.0 (0.6–396.0)	**<0.001**	AB vs. PCN 0.002; DJ vs. PCN 0.003; AB vs. DJ ns (0.444)
HN Grade (Median)	2 (1–2)	2 (1–3)	3 (1–3)	**<0.001**	**AB vs. PCN < 0.001; DJ vs. PCN < 0.001; AB vs. DJ ns**
WBC (10^3^/µL)	12.3 (6.2–29.0)	12.3 (2.8–33.5)	12.5 (6.2–32.1)	0.888	—
Urea (mg/dL)	35.0 (11.2–57.1)	38.2 (4.9–553.0)	42.4 (14.0–255.0)	0.008	AB vs. PCN 0.008; others ns
Temperature (°C)	37.7 (36.0–39.1)	37.9 (35.7–39.8)	38.1 (36.8–39.2)	0.010	AB vs. PCN 0.020; DJ vs. PCN 0.033; AB vs. DJ ns

**Table 2:** Admission-Based Clinical Score (AP-Score) and Risk Stratification.

(A) Score Construction						
Variable		Category		Points		
Hydronephrosis grade		Grade 1		0		
		Grade 2		2		
		Grade 3		4		
CRP at admission (mg/L)		<50		0		
		50–150		1		
		>150		2		
Serum creatinine (mg/dL)		<1.2/1.2–2.0/>2.0		0/1/2		
Total AP-Score				0–8		
**(B) Score-Based Treatment Distribution**						
**Score Band**	**n**	**AB (%)**	**DJ (%)**	**PCN (%)**	***p*** **Value**	**Clinical Interpretation**
0–1 (Low)	37	40.5	59.5	0.0	**<0.001**	No PCN observed
2–3 (Intermediate)	79	19.0	68.4	12.7		DJ most frequent
≥4 (High)	115	6.1	43.5	50.4		PCN most frequent

Dichotomized at the ≥4-point threshold, the nephrostomy rate was 50.4% (58/115) versus 8.6% (10/116) below the threshold (odds ratio 10.79, 95% CI 5.12–22.70, *p* < 0.001; sensitivity 85.3%, specificity 65.0%, negative predictive value 91.4%).

**Table 3:** Final Multivariable Logistic Regression Model Predicting Percutaneous Nephrostomy Use.

Variable	Odds Ratio (OR)	95% Confidence Interval	*p*-Value
Hydronephrosis grade	7.50	4.03–13.96	<0.001
CRP category	2.48	1.58–3.88	<0.001
Creatinine category	1.40	0.84–2.34	0.199

Abbreviations: OR, odds ratio; CI, confidence interval; CRP, C-reactive protein.

**Table 4:** Discriminative Performance of Competing Models and DeLong Comparisons.

Model	AUC (95% CI)	ΔAUC vs. Hydronephrosis Alone	DeLong *p*
AP-Score (HN + CRP + creatinine)	0.830 (0.771–0.886)	+0.058	0.010
Hydronephrosis grade alone	0.772 (0.706–0.832)	reference	—
HN + CRP (without creatinine)	0.826 (0.768–0.876)	+0.054	0.004
Hydronephrosis grade alone (ref.)	0.772	—	—

AUC, area under the receiver operating characteristic curve. DeLong test for correlated ROC curves. AP-Score bootstrap-corrected AUC = 0.827 (1000 resamples); Hosmer–Lemeshow *p* = 0.342.

**Table 5:** Association of the AP-Score with Additional Clinical Outcomes, Adjusted for the Treatment Modality Actually Delivered.

Outcome/Predictor	Effect per 1 AP-Score Point	95% CI	*p*-Value
**Length of stay (days)**	+0.70	0.44 to 0.96	<0.001
Treatment group: DJ	−0.33 days	—	0.621
Treatment group: PCN	+1.74 days	—	0.034
**Time to fever resolution (h)**	+4.76	2.65 to 6.87	<0.001
Treatment group (DJ/PCN)	not significant	—	0.195/0.579

Multivariable linear regression; reference treatment group: antibiotics only. The AP score remained independently associated with both outcomes after adjustment for the intervention actually administered.

**Table 6:** Sensitivity Analyses: Stone Characteristics and CRP Threshold Selection.

Analysis	Result	*p*-Value
**A. Stone location (n = 225, 97.4%)**		
Upper tract (pelvis/UPJ/proximal)	40/121 underwent PCN (33.1%)	0.249
Mid ureter	12/36 underwent PCN (33.3%)	
Distal ureter/UVJ	15/68 underwent PCN (22.1%)	
Added to AP-Score (LR test)	AUC 0.818 → 0.825	0.612
**B. Stone size (n = 58, 25.1%)**		
Univariable	Median 10.0 mm (PCN) vs. 8.0 mm	0.016
Adjusted for hydronephrosis + CRP	OR 1.08 per mm (0.94–1.24)	0.291
**C. CRP threshold sensitivity (AUC)**		
<50/50–150/>150 (selected)	0.830	—
Cohort-derived tertiles (34.7/111)	0.825	—
Range across 7 alternative sets	0.816–0.830	—

Hounsfield unit density was not routinely documented in radiology reports and could not be assessed. LR, likelihood ratio.

## Data Availability

The datasets used and/or analyzed during the current study are available from the corresponding author on reasonable request.
